# Survival analysis for sepsis patients: A machine learning approach to feature selection and predictive modeling

**DOI:** 10.1038/s41598-025-05876-3

**Published:** 2025-07-01

**Authors:** Kaida Cai, Xiaofang Yang, Zhengyan Wang, Wenzhi Fu, Hanwen Liu, Fatemeh Mahmoudi

**Affiliations:** 1 School of Public Health, Southeast University, Nanjing, 210009 China; 2 School of Mathematics, Southeast University, Nanjing, 211189 China; 3 Key Laboratory of Environmental Medicine Engineering, Ministry of Education, School of Public Health, Southeast University, Nanjing, 210009 China; 4 Department of Mathematics and Computing, Faculty of Science and Technology, Mount Royal University, Calgary, T3E 6K6 Canada

**Keywords:** Machine learning, Survival analysis, Feature selection, Missing data imputation, Sepsis, Statistics, Infectious diseases

## Abstract

Sepsis is a life-threatening condition that presents substantial challenges to healthcare and pharmacological management due to its high mortality rates and complex patient responses. Accurately predicting patient outcomes is essential for optimizing therapeutic interventions and improving clinical decision-making. This study evaluates the predictive performance of the Cox proportional hazards model and advanced machine learning techniques, such as extreme gradient boosting (XGBoost), gradient boosting machine (GBM), and random survival forests (RSF), in forecasting survival outcomes for sepsis patients. Feature selection methods, including adaptive elastic net (AEN), smoothly clipped absolute deviation (SCAD), minimax concave penalty (MCP), and information gain (IG), were employed to refine model performance by identifying the most relevant clinical features. The results demonstrate that XGBoost consistently outperforms the Cox model, achieving a higher concordance index and demonstrating superior accuracy in handling complex, non-linear clinical interactions. The integration of feature selection further enhanced the machine learning models’ predictive capabilities. These findings emphasize the potential of machine learning techniques to improve outcome prediction and guide personalized treatment strategies, offering valuable tools for critical care settings.

## Introduction

Sepsis is a serious, life-threatening condition caused by a dysregulated immune response to infection, presenting significant challenges in both medical treatment and healthcare management worldwide. It remains one of the leading causes of in-hospital mortality^1^. Precise quantification of sepsis incidence and mortality rates is not only crucial for improving public health strategies but also for optimizing pharmacological interventions and tailoring treatments to individual patients, ultimately enhancing patient outcomes^2,3^. Current estimates are typically derived from hospital management system data, which, while valuable in clinical scenarios, often fail to provide a complete picture of sepsis’s global impact, particularly in relation to variations in treatment regimens^4^. Continuous analysis and monitoring of mortality risks in sepsis patients are essential for the efficient use of healthcare resources and for personalizing drug therapies to ensure that each patient receives the most appropriate treatment plan^5^.

While previous studies on sepsis management have primarily focused on identifying risk factors and refining early detection methods^5,6^, less attention has been directed toward applying advanced statistical models to improve predictions of patient outcomes, particularly in the context of individualized treatment strategies. Traditional models, like Cox regression, often struggle to handle the complexities of clinical data, especially in capturing non-linear relationships and feature interactions^7,8^. To address these limitations, this study integrates machine learning methods, including RSF, GBM, and XGBoost, that are highly effective in managing complex, non-linear data. To further enhance predictive performance without sacrificing interpretability, feature selection methods such as SCAD, adaptive elastic net (AEN), minimax concave penalty (MCP), and information gain (IG) are incorporated within the Cox regression framework. These feature selection techniques allow models to focus on the most critical features, reducing overfitting and potentially improving drug dosing and treatment strategies by identifying key prognostic markers.

Despite these advances, a clear research gap remains. This gap exists in systematically comparing traditional penalized Cox models with modern machine learning methods for sepsis outcome prediction. In particular, few studies have integrated both penalized regression and nonparametric feature selection techniques. These studies have not evaluated their effectiveness in supporting personalized treatment strategies. To address this gap, this study conducts a comprehensive evaluation. It incorporates refined Cox models with SCAD, MCP, AEN, and information gain-based feature selection. The study also includes machine learning approaches such as RSF, GBM, and XGBoost. Our goal is to assess their relative predictive performance. We aim to evaluate their interpretability for sepsis prognosis, with an emphasis on identifying key prognostic features. These features can inform individualized care.

This study provides a thorough evaluation of refined Cox models compared to machine learning approaches, with a specific focus on their potential to improve sepsis prognosis and support personalized treatment strategies. The findings indicate that machine learning models, particularly XGBoost, consistently outperform traditional methods, offering new insights into the factors influencing sepsis outcomes. The integration of penalized and non-parametric feature selection methods, such as IG, highlights the importance of targeted feature selection in boosting predictive accuracy, which could, in turn, result in optimized therapeutic regimens. Ultimately, this study contributes to better clinical management of sepsis by equipping healthcare professionals with more precise predictive tools, leading to informed treatment decisions, optimized drug administration, and improved patient outcomes. To provide context for the modeling strategies employed in this study, the following section reviews related statistical and machine learning methods, as well as recent advances in interpretable predictive modeling.

The rest of this paper is organized as follows. Section 3 reviews related literature and Section 4 describes the dataset. The proposed methodology, including imputation, feature selection, and model implementation, is detailed across Section 5. Section 6 compares the performance of traditional Cox models and modern machine learning methods, with detailed result analysis. The discussions and conclusions are given in Sections 7 and 8. All analyses are conducted using the R programming language.

## Literature review

Censored survival data is frequently analyzed using the Cox proportional hazards model, which has been widely employed across various studies. However, survival data from sepsis patients typically involve high-dimensional covariates, presenting unique challenges for predictive modeling. Effective feature selection strategies are required to address the presence of numerous, often correlated, covariates, which can impact decisions related to drug dosing and treatment adjustments^9,10^. Methods such as Lasso and smoothly clipped absolute deviation (SCAD) are particularly valuable for managing the complexities of large-scale data through penalized regression techniques^11,12^. Penalized methods with the oracle property are especially useful, as they provide selective and unbiased variable identification while maintaining estimator efficiency, potentially leading to more timely and accurate therapeutic decisions^12,13^. Information theory concepts, such as entropy, also play a role in feature selection by quantifying uncertainty and information gain, which can lead to improved drug efficacy through more targeted interventions^14^.

The rise of “big data” has broadened the horizons of survival analysis by introducing computationally intensive models made possible by advances in computing resources^15^. The random survival forests (RSF) model, for example, stands out as an effective tool for addressing non-linear challenges within machine learning frameworks, due to its use of decision tree ensembles^16,17^. Likewise, gradient boosting machine (GBM) and extreme gradient boosting (XGBoost) methods enhance predictive accuracy by iteratively building on weak learners to create strong, predictive ensembles, demonstrating their effectiveness in both classification and regression tasks^18,19^. These gradient boosting approaches, prized for their computational efficiency and predictive accuracy, also have the potential to support personalized medicine by identifying specific patient subgroups that could benefit from tailored treatments or drug regimens^20,21^. In addition to ensemble methods applied to survival analysis, prior work has demonstrated the potential of machine learning in clinical decision support systems across various diseases. Fathima et al. proposed an enhanced heart disease decision support system using RFE-ABGNB, a feature selection and Naive Bayesâ€“based model optimized for interpretability and accuracy^22^. A separate study combined majority voting with a customized deep neural network to further improve clinical diagnosis performance^23^. In the context of diabetes prediction, Mohideen et al. incorporated regression imputation with an optimized Gaussian Naive Bayes classifier to handle missing data and boost prediction robustness^24^. These studies collectively highlight the utility of feature selection and ensemble-based modeling in healthcare applications. Recent studies have also demonstrated the effectiveness of ensemble learning and interpretable feature optimization in other disease contexts, such as Parkinson’s disease. For instance, explainable models that combine boosting techniques with selective features have been successfully applied to improve diagnostic accuracy and transparency in clinical decision-making^25–27^.

Recent studies have explored the application of machine learning methods for predicting sepsis-related mortality. A systematic review and meta-analysis summarized the effectiveness of various machine learning approaches in sepsis outcome prediction, emphasizing the need for interpretable and clinically applicable models^28^. A simplified mortality prediction model has been proposed for intensive care units, aiming to balance accuracy and model simplicity^29^. In addition, the utility of machine learning models, including tree-based ensembles, has been demonstrated for predicting ICU mortality among sepsis patients^30^. These studies collectively underscore the growing interest in leveraging machine learning for sepsis prognosis, providing a relevant context for our comparative evaluation.

## Dataset description

The dataset analyzed in this study comes from the Medical Information Mart for Intensive Care (MIMIC)-III database, which includes critical care data from over 40,000 patients treated in the intensive care units at Beth Israel Deaconess Medical Center^31,32^. The analysis focuses on data from 6,273 patients diagnosed with sepsis, encompassing 122 clinical features. These features represent a wide range of physiological and clinical measurements, providing a thorough understanding of each patient’s health status. Of these, 20 features capture baseline characteristics such as height, weight, age, gender, and ethnicity. These characteristics are crucial for understanding individual differences in drug metabolism and drug response, helping to personalize treatment plans and optimize dosing strategies tailored to each patient’s demographic and physiological profile. The remaining features record clinical data captured on the first day of hospital admission. These include measurements of immune function, such as white blood cell count and immunoglobulin levels, along with electrolyte concentrations, such as serum chloride and sodium. These data are key in assessing a patient’s response to treatment and guide real-time adjustments to drug therapies, particularly in high-risk populations like sepsis patients. Vital signs, including blood pressure, heart rate, and body temperature, also offer critical insights into the patient’s overall clinical condition. These values directly influence decisions related to medication management, especially for drugs affecting cardiovascular or metabolic functions.

## Methods

**Fig. 1:**
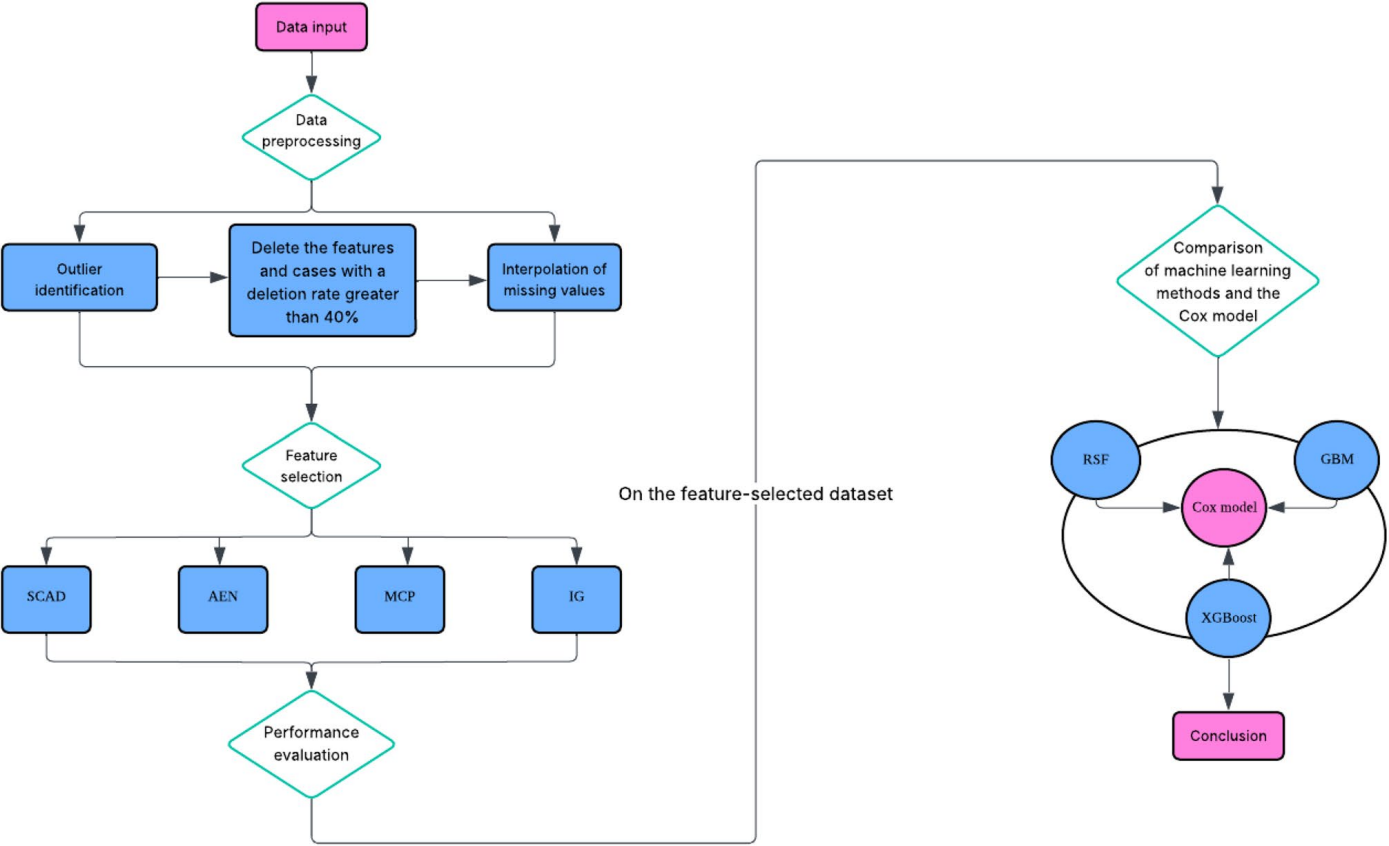
System architecture diagram illustrating the workflow of data preprocessing, feature selection, model training, and performance evaluation.

This study follows a structured workflow encompassing data preprocessing, feature selection, model training, and performance evaluation. To provide an intuitive overview of the entire process, the system architecture diagram is presented in Figure 1.

### Exploratory data analysis

**Fig. 2:**
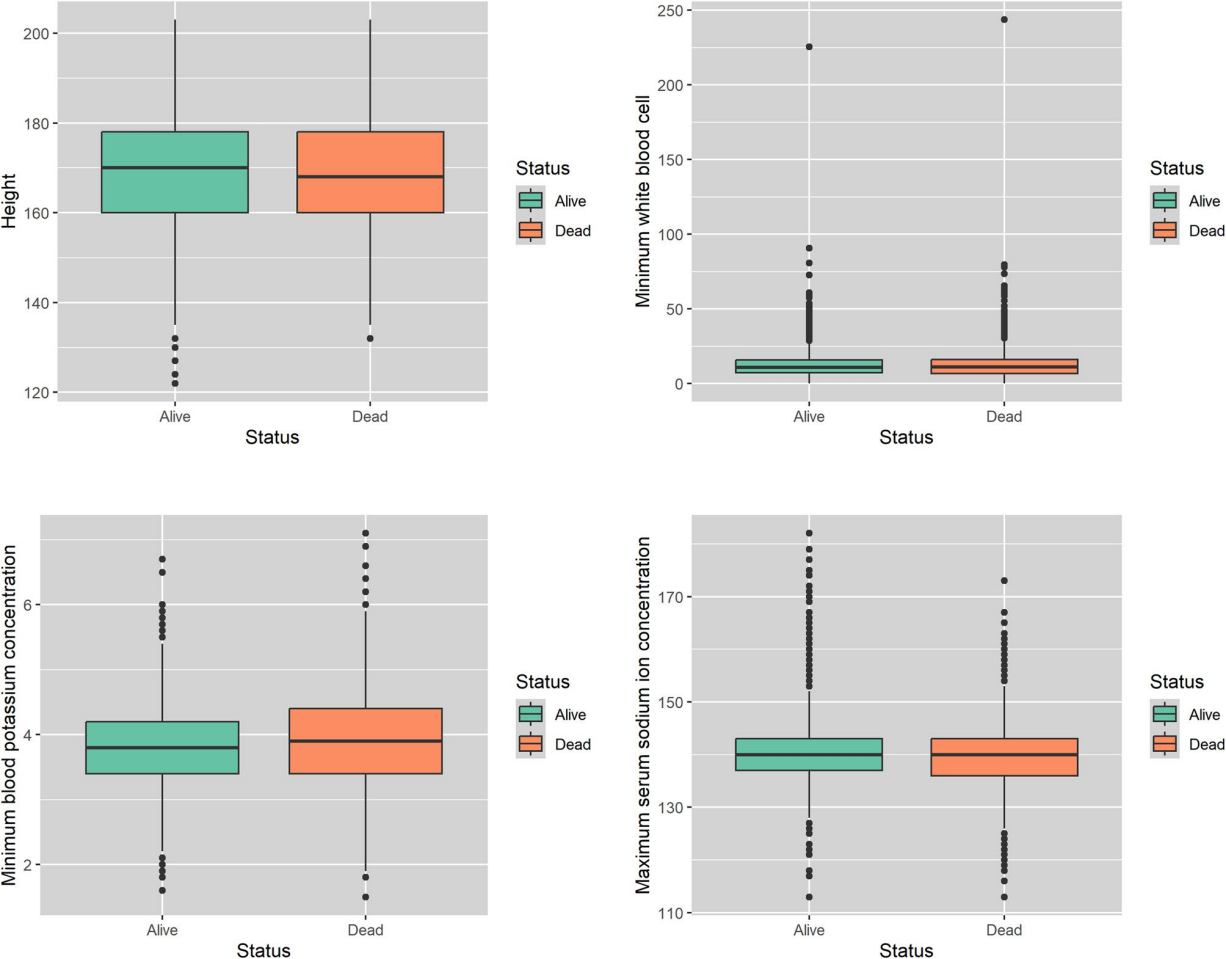
Boxplots showing the values of the four selected features across different survival outcomes.

Table 1 presents summary statistics for four selected features: height, minimum white blood cell count, minimum blood potassium concentration, and maximum serum sodium ion concentration. These features are selected to represent a range of physiological and biochemical factors that play important roles in drug metabolism, immune response, and electrolyte balance. For instance, white blood cell count is a key indicator of immune system activity and helps in determining appropriate dosages for immunomodulatory drugs. Similarly, serum potassium and sodium levels are vital in managing electrolyte imbalances, which are often worsened by medication use in critical care settings. Figure 2 shows boxplots of the four selected features for patients who survived and those who did not. These visualizations demonstrate the variability in these measurements and their potential influence on treatment outcomes. This underscores the importance of individualized treatment strategies, as patients with different physiological characteristics may respond differently to standard drug protocols. The results highlight the need for precision medicine approaches, particularly in sepsis treatment, where accurate and timely adjustments to drug dosing can significantly impact patient outcomes.

**Table 1:** Summary statistics of the four selected features.

	height	wbc.min	bpc.min	ssic.max
Min	122.0	0.1	1.5	113.0
1st Qu	160.0	6.9	3.4	136.0
Median	170.0	11.0	3.8	140.0
Mean	168.8	12.4	3.9	139.9
3rd Qu	178.0	15.9	4.3	143.0
Max	203.0	243.6	7.1	182.0

The table provides the minimum (Min), first quartile (1st Qu), median, mean, third quartile (3rd Qu), and maximum (Max) values for each feature. The abbreviations are as follows: height refers to Height, wbc.min stands for Minimum White Blood Cell Count, bpc.min represents Minimum Blood Potassium Concentration, and ssic.max indicates Maximum Serum Sodium Ion Concentration.

In this study, survival time serves as the response variable, with survival status coded as 1 for patients who died during the study period and 0 for those who survived or were lost to follow-up. This approach allows for a detailed analysis of how different clinical features affect survival and treatment effectiveness. The analysis aims to identify key biomarkers that can inform drug dosing and treatment customization, improving patient outcomes in critical care settings.

### Missing data imputation

Managing missing data is a common obstacle in complex datasets, often arising due to various unpredictable factors^33^. Handling missing data appropriately is particularly important in the field of Pharmaceutics, where accurate and complete patient data are crucial for determining the efficacy and safety of drug therapies^34^. Missing or erroneous data can obscure the understanding of drug interactions, metabolism, and patient responses, which are essential for pharmacokinetic and pharmacodynamic modeling. To address this issue, we employed the classification and regression trees (CART) approach for imputation, implemented via the MICE package in R. CART is a flexible, non-parametric method capable of handling both continuous and categorical features. It captures nonlinear relationships and complex feature interactions, making it well suited for complex clinical data with mixed variable types and missingness patterns^35^. The algorithm builds decision trees by recursively partitioning the data based on features that maximize information gain, thereby reducing uncertainty in the imputation process. Missing values are then estimated using the observed data from similar instances within the tree structure, preserving the internal relationships of the dataset. CART was selected over alternative imputation methods such as predictive mean matching or k-nearest neighbors (KNN) due to its robustness to outliers, ability to model nonlinearity, and empirical stability in clinical settings. While the MICE framework supports multiple imputation strategies, we chose CART as the base learner because it aligns well with the data complexity and feature dependencies present in our sepsis cohort.

### Feature selection

It is crucial in practical applications to meticulously select significant features from a vast array of potential factors to ensure accurate assessments of their impacts^13^. Indiscriminate inclusion of all features can increase computational demands and extend model training durations, which may lead to inefficient use of resources. Additionally, constraints in data collection might result in the inclusion of irrelevant or conflicting factors, potentially undermining the model’s accuracy and reliability. An excessive inclusion of features also risks overfitting, which could degrade the model’s predictive performance. This highlights the critical role of feature selection in optimizing the Cox model. To manage this, penalized feature selection is commonly applied to restrain the coefficients in the Cox model, effectively reducing the number of features while boosting its predictive strength^12^. Based on the log-partial likeihood function (5), the objective function of the penalized Cox regression method can be described as follows

1 $$\begin{aligned} Q(\beta ) = \ell (\beta ) - \sum _{j=1}^{p} P_{\lambda } (\beta _j). \end{aligned}$$

By maximizing this objective partial function, the model allows for efficient selection of significant features, enhancing the interpretability and predictive power of survival analysis, especially in medical research and related fields.

While traditional penalization methods such as LASSO and Ridge regression have been widely used in Cox models, they may suffer from limitations in handling correlated variables or biased estimation. Therefore, in this study, we adopt three more advanced alternatives for feature selection within the Cox regression framework, including smoothly clipped absolute deviation (SCAD), adaptive elastic net (AEN), and minimax concave penalty (MCP). In addition to the penalized feature selection methods, we also incorporate information gain (IG) as a feature selection criterion. Information gain measures the reduction in entropy, or uncertainty, of the outcome feature when a feature is used to split the data^14^. For a given feature $X_j$, the information gain is defined as

2 $$\begin{aligned} I(X_j) = G(Y) - G(Y | X_j), \end{aligned}$$

where $G(Y)$ is the entropy of the outcome feature $Y$, and $G(Y | X_j)$ is the conditional entropy of $Y$ given the feature $X_j$. Entropy $G(Y)$ is calculated as

3 $$\begin{aligned} G(Y) = - \sum _{k=1}^{K} p_k \log p_k, \end{aligned}$$

where $p_k$ is the probability of outcome $k$, and $K$ is the number of possible outcomes. Information gain evaluates how much knowing the feature $X_j$ reduces the uncertainty of predicting $Y$, with higher values indicating more informative features. By integrating information gain into the feature selection process, we can identify the most relevant features that contribute to survival prediction. This method complements the penalized approaches by offering a non-parametric way of assessing feature importance, enhancing both the interpretability and the predictive power of the Cox model in high-dimensional settings. These feature selection methods and the Cox model are integrated to develop three specialized methods: Cox-SCAD, Cox-AEN, Cox-MCP and Cox-IG.

### Cox proportional hazards model

#### Model overview

The Cox proportional hazards model, often referred to as the Cox model, is a commonly used statistical tool for analyzing survival data^36^. It is semi-parametric, meaning it does not make specific assumptions about the distribution of survival times. Instead, it models the hazard function, which is expressed as

4 $$\begin{aligned} h(t,X) = h_0(t) \exp (\beta _1X_1 + \beta _2X_2 + \ldots + \beta _pX_p), \end{aligned}$$

where $t$ represents survival time, $h(t,X)$ is the hazard function conditional on covariates $X_1, X_2, \ldots , X_p$, and $\beta _1, \beta _2, \ldots , \beta _p$ are the coefficients that quantify the effect of these covariates. The term $\exp (\beta _1 X_1 + \ldots + \beta _p X_p)$ captures the multiplicative effect of the covariates on the baseline hazard. The baseline hazard $h_0(t)$ represents the hazard assuming all covariates are zero. The flexibility of this model lies in the fact that the baseline hazard $h_0(t)$ is unspecified, allowing it to handle a variety of survival data. The Cox model assumes that the hazard ratio between any two individuals remains constant over time, known as the proportional hazards assumption. This assumption facilitates comparisons between different groups in survival analysis.

#### Parameter estimation

Let $\delta _i \in \{0, 1\}$ be an indicator of whether the event of interest (e.g., death or failure) is observed ($\delta _i = 1$) or censored ($\delta _i = 0$). To estimate the regression coefficients $\beta$, the Cox model relies on maximizing the log-partial likelihood, which is defined as

5 $$\begin{aligned} \ell (\beta ) = \sum _{i: \delta _i = 1} \left( \beta ^\top X_i - \log \left( \sum _{j \in R(t_i)} e^{\beta ^\top X_j} \right) \right) , \end{aligned}$$

where $R(t_i)$ denotes the risk set at time $t_i$, i.e., the set of individuals still under observation (at risk) just prior to time $t_i$. The set $R(t_i)$ refers to the individuals at risk at time $t_i$. By maximizing this log-partial likelihood, the estimates of $\beta$ are obtained, providing insights into how each covariate affects the hazard function.

The log-partial likelihood $\ell (\varvec{\beta })$ is concave in $\varvec{\beta }$, and its maximization yields the maximum partial likelihood estimates of the regression coefficients. These estimates are typically computed using iterative numerical procedures, such as the Newton-Raphson or Fisher scoring algorithm. The interpretation of each coefficient $\beta _j$ is as the log hazard ratio associated with a one-unit increase in the corresponding covariate $X_j$, holding all other covariates constant. That is, $\exp (\beta _j)$ represents the hazard ratio for a one-unit increase in $X_j$.

### Machine learning methods

In this study, we apply several machine learning techniques to datasets optimized through effective feature selection and missing data imputation strategies. The goal is to assess and compare the predictive performance of traditional Cox models alongside various machine learning methods, including random survival forests (RSF), the gradient boosting machine (GBM), and extreme gradient boosting (XGBoost). These techniques effectively handle complex survival data, capture non-linear relationships, and do not rely on the proportional hazards assumption. RSF accommodates censored data and complex interactions, while GBM and XGBoost offer strong predictive performance through iterative boosting and advanced regularization^16–19^. Their proven robustness and flexibility make them well-suited for modeling sepsis outcomes. To ensure the robustness and reliability of the analysis, we employ ten-fold cross-validation, where the dataset is partitioned into ten equal subsets. Each subset is used once as the validation set, while the remaining nine serve as the training set, allowing for comprehensive model evaluation across the entire dataset.

#### Random survival forests

The random survival forests method is used to analyze right-censored survival data by constructing survival trees and estimating cumulative hazard functions^16,17^. The algorithm proceeds in three main steps:

1. Bootstrap Sampling: For each tree in the ensemble, approximately 63% of patients are randomly selected with replacement from the original dataset to form a bootstrap sample. Some patients may appear more than once. The remaining 37% of patients, those not selected, constitute the out-of-bag (OOB) set. This OOB data serves as an internal validation subset for estimating prediction error without requiring a separate test set.

2. Survival Tree Construction: A survival tree is grown on each bootstrap sample. At each node, a random subset of features is considered for splitting. Splits are selected to maximize survival differences using log-rank statistics. The tree continues to grow until a pre-specified minimum number of events is reached in the terminal nodes.

3. Ensemble Prediction: Each survival tree produces a cumulative hazard function (CHF). The final ensemble prediction is obtained by averaging the CHFs from all trees. Predictions for the OOB samples, which were not used in building their respective trees, provide an unbiased estimate of model performance.

RSF eliminates the proportional hazards assumption, allowing it to capture complex non-linear relationships in sepsis data more effectively. Its ability to model complex clinical data is enhanced by feature randomization at each split. The use of bootstrap aggregation improves robustness by reducing variance, while the OOB framework enables internal validation. Additionally, preprocessing of extreme lab values prior to model training helps mitigate sensitivity to outliers.

#### Gradient boosting machine

The gradient boosting machine is a powerful ensemble learning method that builds predictive models by combining a sequence of simpler models, referred to as weak learners, which are typically decision trees^18^. The process begins with the creation of an initial model, often based on basic statistics such as the mean or median of the target variable. As the method progresses, each subsequent model focuses on correcting the prediction errors made by the previous models. These weak learners are trained to minimize the residuals, or differences, between the observed outcomes and the predictions made so far. In each iteration, a new model is added to the ensemble, specifically trained to address the errors made by the models that came before. The combined effect of this iterative process is that the overall predictive accuracy improves with each new model. The key factor controlling the strength of these iterative corrections is the learning rate, which ensures that each model’s contribution is carefully scaled to avoid overfitting. This incremental improvement helps the model gradually refine its predictions, providing a more accurate representation of the underlying data.

One of the distinguishing characteristics of GBM is its focus on minimizing residual errors through sequential updates. Unlike methods such as random survival forests, which generate multiple models independently and then average their predictions, GBM trains each model to directly address the errors left by the preceding models. This makes it particularly effective at handling complex datasets, as the sequential adjustments allow for a more refined and targeted improvement in predictive performance.

#### Extreme gradient boosting

Extreme Gradient Boosting is an advanced ensemble learning technique that has been optimized for both high performance and computational efficiency, making it a popular choice in data science^19^. In the context of survival analysis, XGBoost has been adapted to handle censored data, which is commonly found in survival outcomes^37^. This adaptation allows XGBoost to account for incomplete survival information, ensuring that the model can effectively predict outcomes even when some data points are censored. XGBoost operates by iteratively building a series of regression trees, each of which refines the predictions made by the previous ones. Unlike standard regression trees, the objective function used in XGBoost includes a regularization term that penalizes model complexity, helping to prevent overfitting. This regularization term is particularly useful in survival analysis, where the model must balance predictive accuracy with the need to generalize well to new, unseen data.

Each iteration of XGBoost works by adjusting the model based on the errors made by earlier iterations. These adjustments are carefully scaled to ensure that the model improves incrementally without becoming too complex. Additionally, XGBoost incorporates techniques designed specifically for survival analysis, such as methods for dealing with censored data, ensuring that it can effectively model time-to-event outcomes while accommodating the incomplete nature of survival data. One of the strengths of XGBoost is its ability to handle large datasets and complex models efficiently, while also providing mechanisms to prevent overfitting. This makes it particularly well-suited for survival analysis in areas like medical research, where the data can be both high-dimensional and censored. By incorporating both predictive accuracy and regularization, XGBoost offers a robust framework for survival analysis that performs well even in challenging scenarios.

All three machine learning models used in this study are equipped to handle right-censored survival data through their respective objective functions or splitting mechanisms. RSF applies a log-rank splitting rule that compares survival distributions across nodes using only individuals still at risk at each event time. This approach allows the model to properly account for censoring during tree construction. GBM is implemented with a Cox proportional hazards loss function, where the optimization is based on the Cox partial likelihood. This formulation inherently adjusts for censoring by using risk sets, similar to the classical Cox model. XGBoost is used with the survival:cox objective, which optimizes the negative partial log-likelihood of the Cox model. Censored observations are excluded from the event contribution but included in the risk sets, ensuring accurate handling of censored data during training. Together, these methods offer robust solutions for modeling survival data with censoring, enabling fair and meaningful comparisons with the Cox proportional hazards model. For each machine learning model, the key hyperparameters are selected using a combination of empirical defaults and cross-validation. Specifically, 10-fold cross-validation on the training data is employed to guide tuning decisions, ensuring a balance between model complexity and generalization performance. This strategy avoids overfitting while maintaining computational efficiency and is consistent with practices commonly adopted in the survival analysis literature.

### Performance metrics

To evaluate the performance of methods developed through integrating the Cox regression framework with various penalties and missing data imputation techniques, we utilize several performance metrics specifically tailored for survival data. These include time-dependent accuracy (ACC), time-dependent area under the curve (AUC), and receiver operating characteristic (ROC) curves to assess the results of feature selection^38^. Additionally, ACC, AUC, and the concordance index (C-index) are employed to compare the predictive accuracy of the machine learning methods and Cox model based on the analyzed datasets. Specifically, we note that the C-index evaluates the concordance between predicted and observed event times, but may be biased when the number of events is small relative to censored cases. Similarly, time-dependent AUC values can be influenced by skewed risk distributions and should be interpreted with caution under imbalance. To assess the degree of class imbalance in the outcome variable, we calculated the proportion of censored and event cases. Among the 5,895 patients included in the final dataset, 60.1% were censored (status = 0) and 39.9% experienced the event (status = 1), indicating a mild level of imbalance. As this distribution is commonly observed in clinical survival datasets and does not constitute a severe imbalance, we do not apply resampling or weighting techniques.

The **time-dependent accuracy**, denoted as ACC$(t)$, measures how accurately the model classifies individuals as likely or unlikely to experience the event (e.g., death) by a specific time point. For instance, in a clinical dataset aimed at predicting 3-year survival, a high ACC at year 3 would suggest that the model reliably identifies patients at high risk of dying within that time frame based on their covariate profiles. ACC$(t)$ is the proportion of correctly classified individuals at a given time $t$, accounting for censoring. It is computed as:

6 $$\begin{aligned} \text {ACC}(t) = \frac{\text {TP}(t) + \text {TN}(t)}{\text {TP}(t) + \text {TN}(t) + \text {FP}(t) + \text {FN}(t)}, \end{aligned}$$

where $\text {TP}(t)$ and $\text {TN}(t)$ are the true positives and true negatives at time $t$, while $\text {FP}(t)$ and $\text {FN}(t)$ are false positives and false negatives, respectively. In the context of our study, ACC provides a direct measure of how well the selected features contribute to classifying patients at specific survival horizons.

The **time-dependent AUC** and the associated ROC curves evaluate the model’s ability to distinguish between individuals who experience the event before a given time and those who survive beyond it. For example, an AUC at 5 years of 0.85 for a model with SCAD penalization and imputed data would indicate strong discrimination between high- and low-risk patients at that time point. These metrics help assess whether penalization and imputation strategies improve the model’s classification capability. Time-dependent AUC was computed using the timeROC R package, focusing on 30-day survival as a clinically meaningful endpoint. The 30-day window is widely adopted in sepsis studies to capture acute-phase mortality^39^. ROC curve plots the true positive rate (TPR) against the false positive rate (FPR). These are defined as:

7 $$\begin{aligned} \text {TPR}(t)&= \frac{\text {TP}(t)}{\text {TP}(t) + \text {FN}(t)}, \end{aligned}$$

8 $$\begin{aligned} \text {FPR}(t)&= \frac{\text {FP}(t)}{\text {FP}(t) + \text {TN}(t)}. \end{aligned}$$

A higher AUC indicates improved separation between individuals who experience the event before time $t$ and those who do not. In our study, we use AUC to evaluate how well imputed and penalized models distinguish between high- and low-risk patients at clinically relevant time points.

In evaluating survival models, the concordance index (C-index) remains a key metric. The **C-index** measures the global ranking ability of the model across all survival times. It measures how well the model’s predicted survival times (or risk scores) correspond with actual outcomes by comparing pairs of individuals. The concordance index is calculated as

9 $$\begin{aligned} C\text {-}index = \frac{\sum _{(i,j) \in \Omega } I\{{\hat{s}}_i < {\hat{s}}_j\} + 0.5 \times I\{{\hat{s}}_i = {\hat{s}}_j\}}{|\Omega |}, \end{aligned}$$

where $I$ is an indicator function, $\Omega$ represents all comparable pairs of individuals, and ${\hat{s}}_i$ and ${\hat{s}}_j$ are the predicted survival probabilities or risk scores for individuals $i$ and $j$, respectively. A concordance index of 1 indicates perfect concordance, while 0.5 indicates random prediction, and values above 0.7 generally suggest good predictive accuracy^40^. For example, if a patient predicted to have higher risk dies before one predicted with lower risk, that pair is concordant. The C-index is particularly valuable in our context for comparing the overall predictive accuracy of models incorporating different feature sets and imputation strategies, especially under heavy censoring. By leveraging these metrics, we systematically assess both.

These metrics, including ACC, AUC, ROC curves, and C-index, provide a comprehensive framework for evaluating the effectiveness of feature selection methods and comparing the predictive performance of survival models built using the imputed and optimized datasets.

## Results

### Missing data and imputation

**Fig. 3:**
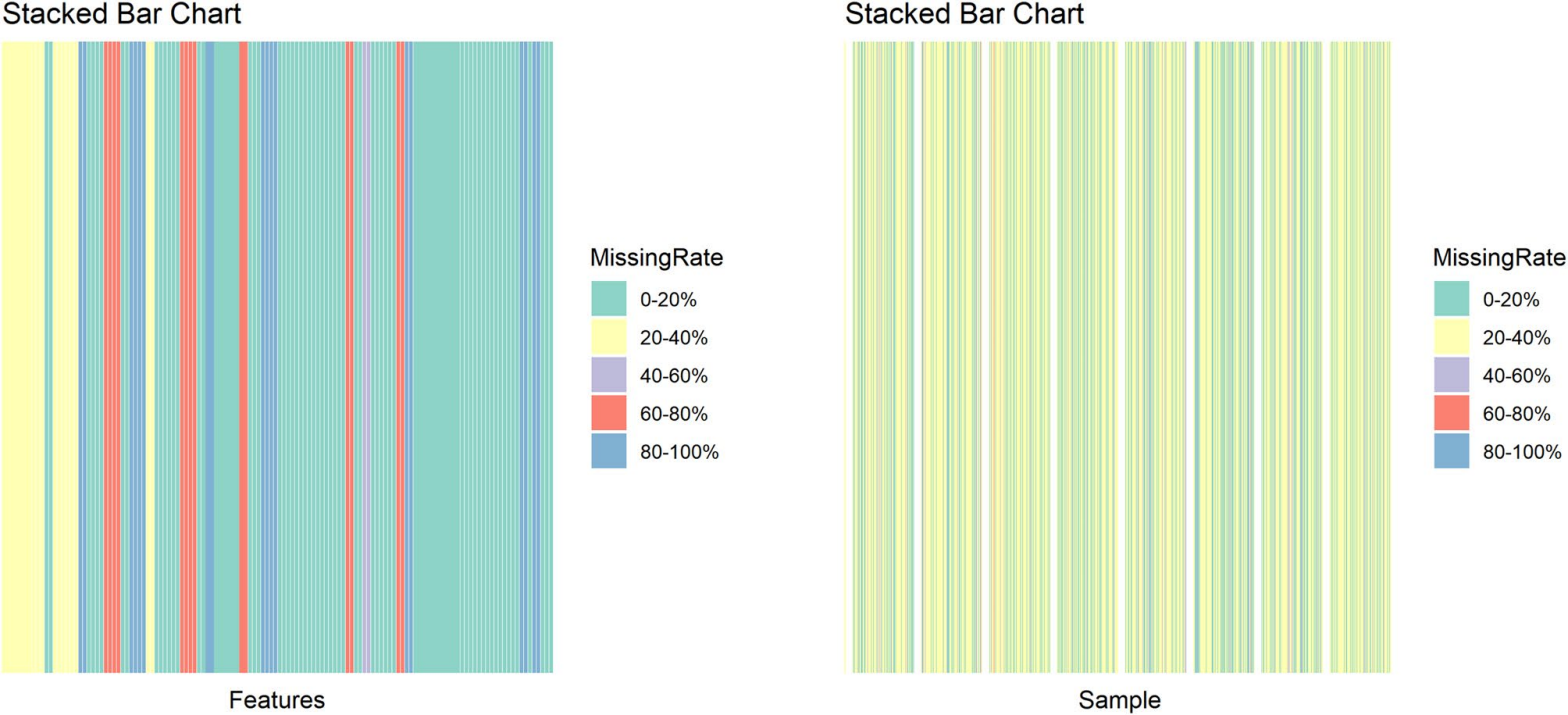
Stacked bar charts illustrating the distribution of missing data across features and samples in the dataset, categorized by different missing rate intervals.

The dataset shows a significant amount of missing data and includes outliers, especially in features like patient weight and hospital stay duration. To maintain analytical robustness, these outliers were treated as missing values. The distribution of missing data across the dataset is visualized in Figure 3, with a more detailed breakdown of missing values provided in Table 2. Specifically, 34 features and 378 cases exhibit a missing rate greater than 40%. To improve the reliability and accuracy of the statistical analysis, we removed features and cases with a missing rate above this threshold. To handle missing data, we applied the CART imputation method implemented in the mice R package. The imputation was conducted using method “cart” with a fixed random seed (seed = 1234) to ensure reproducibility. Among the five imputed datasets generated by default, the third completed dataset was selected using the “complete()” function for further analysis.

**Table 2:** Summary of missing rates across features and samples, categorized by different intervals of missing data.

Interval of missing rate	Feature		Sample	
	Frequency	Percentage(%)	Frequency	Percentage(%)
[0,0.2]	59	53.15	2128	33.92
(0.2,0.4]	18	16.22	3767	60.05
(0.4,0.6]	2	1.80	368	5.87
(0.6,0.8]	14	12.61	10	0.16
(0.8,1]	18	16.22	0	0

**Fig. 4:**
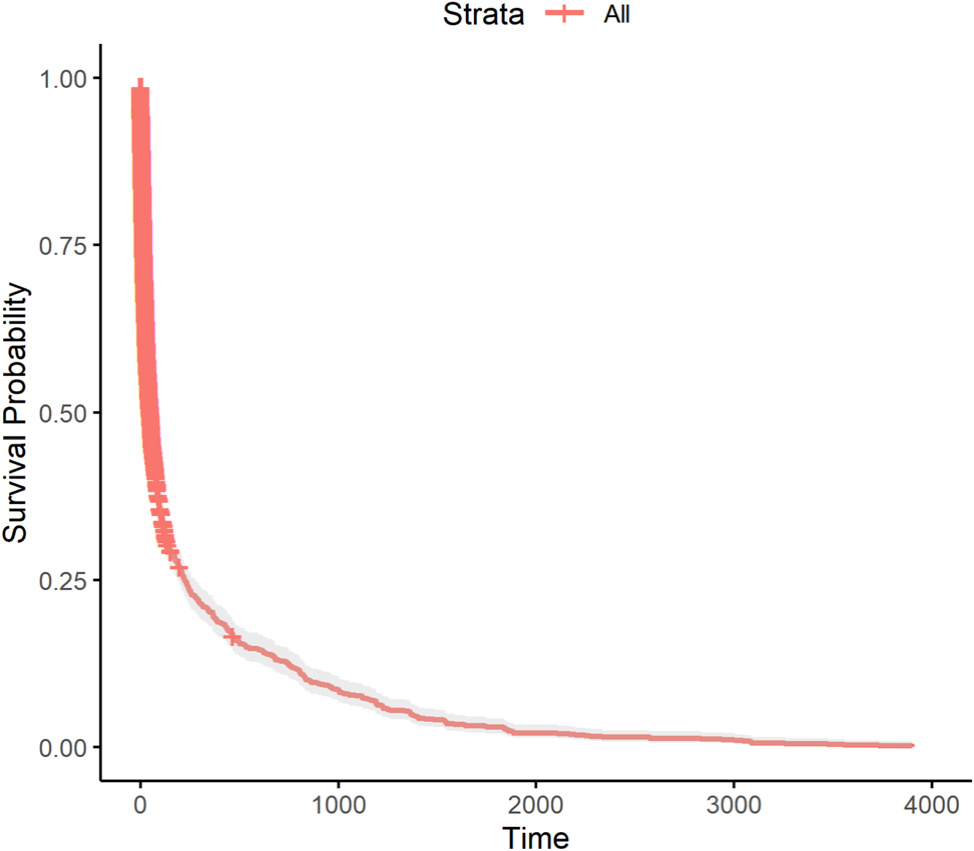
The overall survival curve of 5895 samples.

After imputing missing values in the original dataset, we retain 5,895 samples and 88 features. The Kaplan-Meier survival curve displayed in Figure 4 provides an overall view of the survival probabilities across the entire cohort. The curve shows a rapid drop in survival probability early on, followed by a more gradual decline as time progresses. After an extended period, the chances of survival become very low, eventually approaching zero. This pattern indicates a high mortality rate early on, followed by a gradual leveling off among the remaining individuals. The plot further illustrates the broad survival trends in the cohort without stratification by specific features or subgroups. The shaded region around the curve reflects the confidence interval, providing a visual representation of the uncertainty in the estimates. These results suggest that, while a substantial portion of the cohort experiences early mortality, a smaller group exhibits more prolonged survival. This emphasizes the need for further analysis to identify the features that are most predictive of longer survival times.

### Feature selection and interpretation

In this study, several key categorical features, such as gender, ethnicity, and clinical history, are encoded as dummy features, while numerical features are standardized to improve model performance. Given the importance of these features in pharmacotherapy, especially in understanding how demographic and physiological factors influence drug metabolism and efficacy, this approach ensures that the models capture essential patient-specific information. The dataset is then divided into a training set (80%) and a test set (20%) to evaluate the predictive models robustly. Feature selection is a crucial step in refining predictive models, particularly in pharmacological research, where identifying the most relevant clinical features leads to more precise and personalized treatment regimens. Four feature selection methods, inlcuding adaptive elastic net (AEN), smoothly clipped absolute deviation (SCAD), minimax concave penalty (MCP), and information gain (IG), are employed. These methods help identify features that are most predictive of survival outcomes, which, in turn, have implications for optimizing drug dosages, monitoring therapeutic efficacy, and understanding pharmacodynamics in diverse patient populations.

Table 3 summarizes the results of feature selection. IG selects the most features, identifying 53 features, which include important clinical and biochemical parameters such as minimum white blood cell count, serum sodium concentration, and other biomarkers that are often used to guide pharmaceutical interventions. AEN follows closely by selecting 47 features, including vital signs such as body temperature and heart rate, which are both crucial indicators for adjusting drug dosages and evaluating patient responses to treatment. SCAD, a more conservative method, selects 32 features, focusing on critical factors like blood glucose levels and systolic blood pressure. MCP, the most selective method, chooses only 26 features, but these features, such as platelet count and lactate levels, are particularly significant in assessing coagulation status and metabolic function, which are vital for guiding therapeutic interventions in critically ill patients.

**Table 3:** Feature selection results of four feature selection methods, represented by the number of selected features.

Penalty	No. of Features
AEN	47
SCAD	32
MCP	26
IG	53

**Fig. 5:**
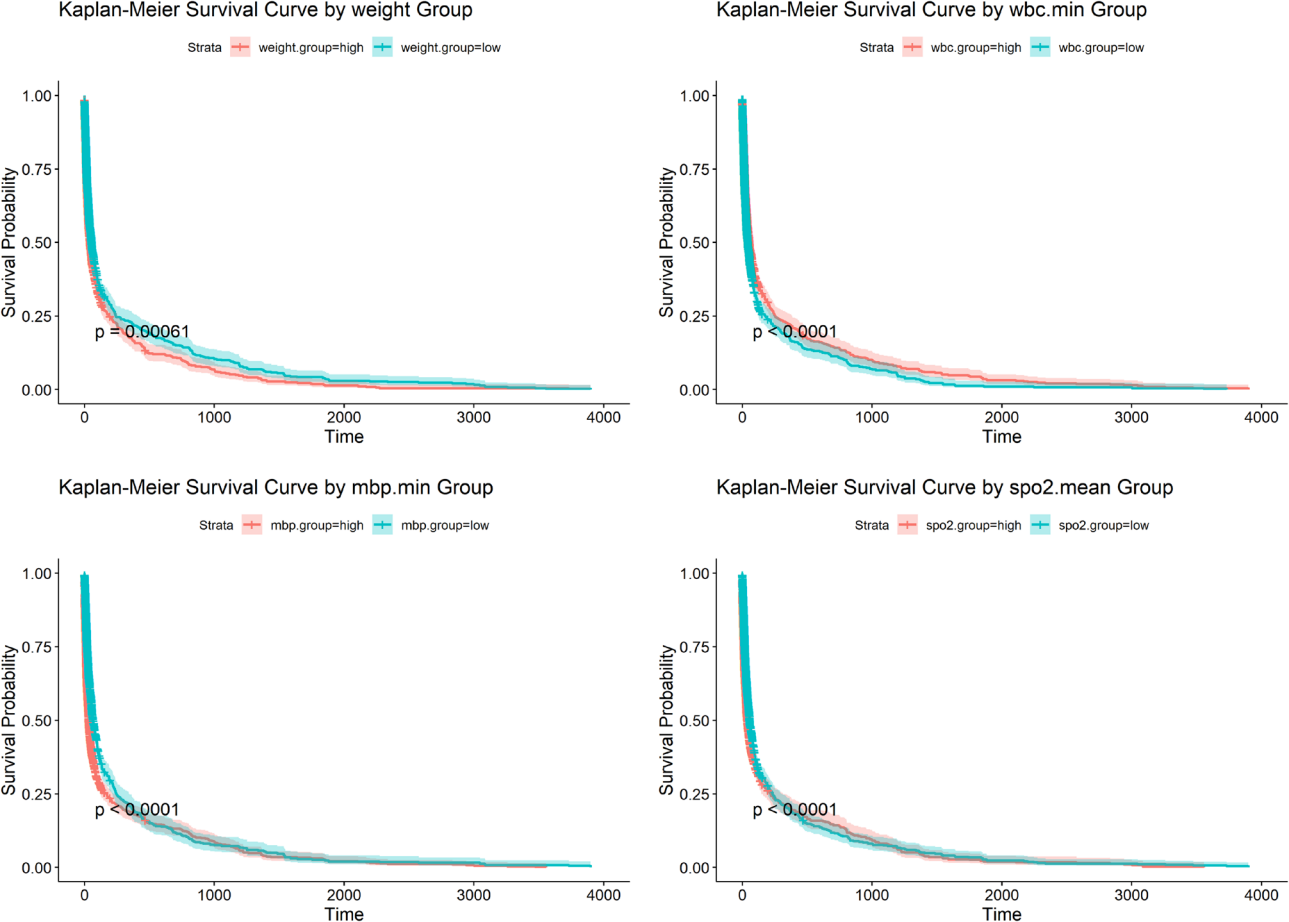
Kaplan-Meier survival curves for four key features: Weight, Minimum White Blood Cell Count (wbc.min), Minimum Blood Pressure (mbp.min), and Mean Blood Oxygen Saturation (spo2.mean). These curves illustrate the survival differences between high and low groups for each feature, providing insights into their potential impact on drug metabolism and therapeutic response.

The selected features include several that are particularly relevant to drug metabolism and pharmacokinetics in sepsis patients. For instance, white blood cell count is a key marker of immune response and guides the administration of immunomodulatory therapies. Serum sodium and potassium levels are critical for managing fluid and electrolyte imbalances, often exacerbated by drug treatments, especially in critically ill patients. By focusing on these biomarkers, the feature selection methods provide valuable insights into optimizing treatment regimens and personalizing pharmaceutical interventions. Notably, there is significant overlap in the features selected by the various methods, with approximately 15 key features consistently chosen. These include features such as weight, minimum white blood cell count, minimum blood pressure, and mean blood oxygen saturation, all of which are critical for tailoring drug therapies to the individual patient. These features are further analyzed using Kaplan-Meier survival curves to evaluate their impact on patient outcomes, as shown in Figure 5. The Kaplan-Meier curves for weight, white blood cell count, blood pressure, and blood oxygen saturation reveal significant differences in survival between high and low groups for each feature. For example, patients with lower body weight often have reduced drug metabolism, which necessitates dosage adjustments to prevent adverse effects. Similarly, lower white blood cell counts are associated with compromised immune function, highlighting the need for targeted immune-modulating therapies. Blood pressure and oxygen saturation are key indicators of cardiovascular and respiratory function, both of which are directly impacted by pharmacological treatments, especially in the context of septic shock. These findings underscore the importance of individualized treatment strategies, particularly in critical care settings where pharmacotherapy must be carefully tailored to the patient’s physiological condition to optimize outcomes.

**Fig. 6:**
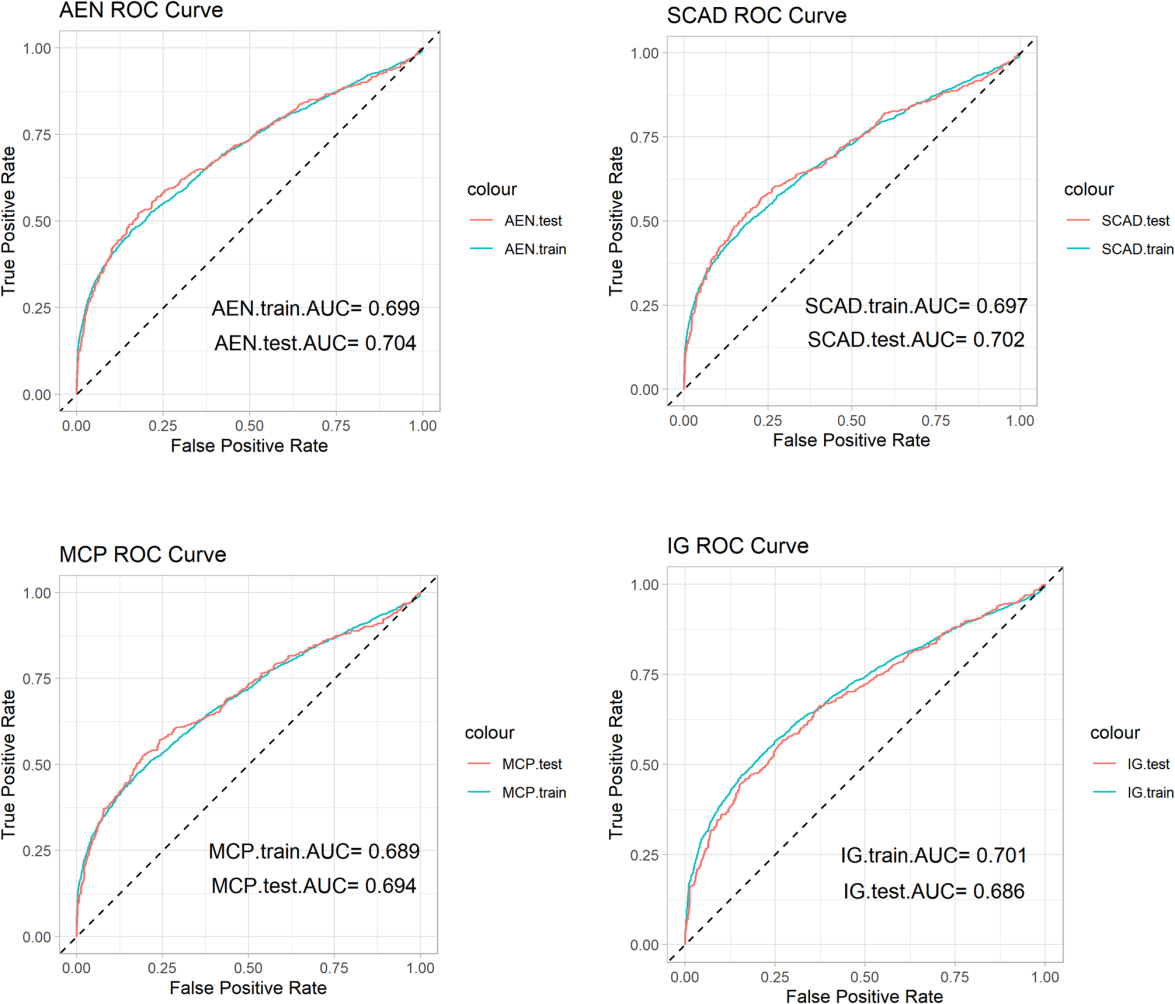
ROC curves and corresponding AUC values for four feature selection methods: AEN, SCAD, MCP, and IG, comparing their performance on training and test data.

**Fig. 7:**
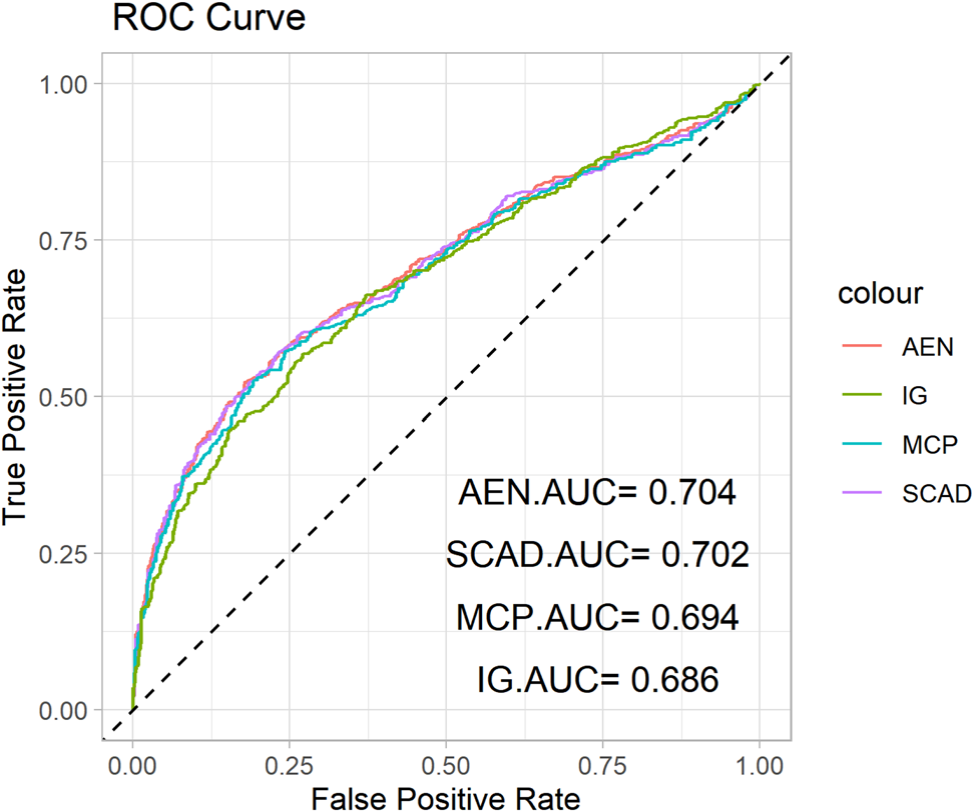
ROC curves and AUC values of four feature selection methods evaluated on the test data.

In the evaluation of the selected features, performance metrics such as the C-index, accuracy, and AUC, or area under the ROC curve, assess the predictive power of the models as illustrated in Figures 6 and 7. Table 4 summarizes these performance metrics, showing that AEN consistently performs well across all metrics, yielding a C-index of 0.822 and an AUC of 0.704. This indicates strong predictive capability, particularly in identifying which patients may benefit most from specific pharmaceutical interventions. SCAD and MCP also demonstrate solid performance, while IG, though selecting the most features, has slightly lower accuracy, suggesting that more focused feature selection methods may be better suited for certain clinical applications.

**Table 4:** C-index, ACC, and AUC values for the test data across different feature selection methods.

Penalty	C-index	ACC	AUC
AEN	0.822	0.707	0.704
SCAD	0.824	0.692	0.702
MCP	0.825	0.699	0.694
IG	0.811	0.668	0.686

Table 5 presents the confusion matrices for the four feature selection methods. It is evident from the results that the AEN method outperforms SCAD, MCP, and IG in terms of accuracy, consistently achieving a better balance between true positives and true negatives. AEN has the most favorable classification results, with fewer false positives and false negatives compared to the other methods. SCAD and MCP, while performing reasonably well, display a slightly less balanced classification. SCAD tends to underclassify positive instances, whereas MCP shows similar tendencies but to a lesser degree. IG, on the other hand, struggles more significantly with true negative classification, resulting in a higher number of false positives, indicating comparatively lower precision. Overall, AEN proves to be the most effective method, consistently showing higher classification accuracy across the dataset. SCAD and MCP also perform well but exhibit more variability, while IG, although useful, lags behind the other methods in terms of classification precision and overall performance.

**Table 5:** Confusion matrices of the four feature selection methods.

Actual $\backslash$Predicted	AEN		SCAD		MCP		IG	
	0	1	0	1	0	1	0	1
0	594	129	556	167	584	139	528	195
1	217	239	196	260	216	240	197	259

In summary, the comparison of feature selection methods highlights the strength of AEN in both feature identification and model performance. AEN consistently achieves higher classification accuracy, as evidenced by its performance metrics and confusion matrix results. SCAD and MCP follow closely, showing reliable yet slightly more conservative feature selection tendencies, while IG, though selecting the most features, demonstrates comparatively lower precision. Overall, these methods, particularly AEN, effectively handle feature selection of survival data, improving the model’s predictive accuracy and enhancing the interpretability of critical clinical features. By tailoring drug regimens based on the key features identified through feature selection, healthcare providers can ensure more effective therapeutic strategies, ultimately leading to better patient outcomes. The incorporation of these methods into pharmaceutical research and clinical practice supports the growing need for precision medicine, especially in critical care settings like sepsis, where timely and accurate treatment adjustments are essential. The selected feature subsets obtained from AEN, SCAD, MCP, and IG are subsequently used as inputs for all predictive models evaluated in this study, including the Cox proportional hazards model, RSF, GBM, and XGBoost. This consistent use of optimized feature sets ensures a fair comparison of model performance and reflects the practical application of feature selection in guiding predictive modeling.

### Model performance comparison

In this study, we evaluate the performance of several machine learning approaches, including random survival forests, gradient boosting machine, and extreme gradient boosting, in comparison to the Cox proportional hazards model, which is widely used in survival analysis. Our objective is to examine the relative effectiveness of these methods, particularly when analyzing survival datasets characterized by complex and non-linear interactions between features. By employing feature selection through the AEN penalized method, we are able to assess how these models perform in predicting survival outcomes, a crucial task in personalized drug therapies and clinical decision-making.

Table 6 summarizes the performance metrics, including C-index, ACC, and AUC, across all methods. Among the machine learning models, XGBoost achieves the highest overall performance, with a C-index of 0.929. This result underscores XGBoost’s capability to model non-linear interactions and capture intricate relationships within the data, surpassing the traditional Cox model, which records a C-index of 0.839. Both RSF and GBM also demonstrate strong performance, with C-index values of 0.883 and 0.919, respectively. These results highlight the strength of ensemble methods like RSF and boosting techniques such as GBM in handling survival data, particularly in scenarios that require precision in predicting patient responses to pharmaceutical treatments.

Further analysis of ACC and AUC values provides additional insights into the strengths of each model. XGBoost achieves an ACC of 0.702, slightly outperforming RSF’s ACC of 0.700. Both models surpass the Cox model’s ACC of 0.699, indicating that these machine learning approaches are more effective in making accurate predictions. Although RSF achieves the highest AUC value at 0.722, demonstrating its ability to distinguish between events and non-events in survival data, XGBoost and the Cox model follow closely with AUC values of 0.694 and 0.699, respectively. GBM, despite its strong performance in terms of C-index, shows slightly lower AUC and ACC values, suggesting that its handling of the feature interactions is less effective than that of RSF and XGBoost in this particular dataset.

**Table 6:** Values of C-index, ACC, and AUC for the Cox model and machine learning methods.

Method	C-index	ACC	AUC
Cox	0.839	0.699	0.699
RSF	0.883	0.700	0.722
GBM	0.919	0.682	0.654
XGBoost	0.929	0.702	0.694

**Fig. 8:**
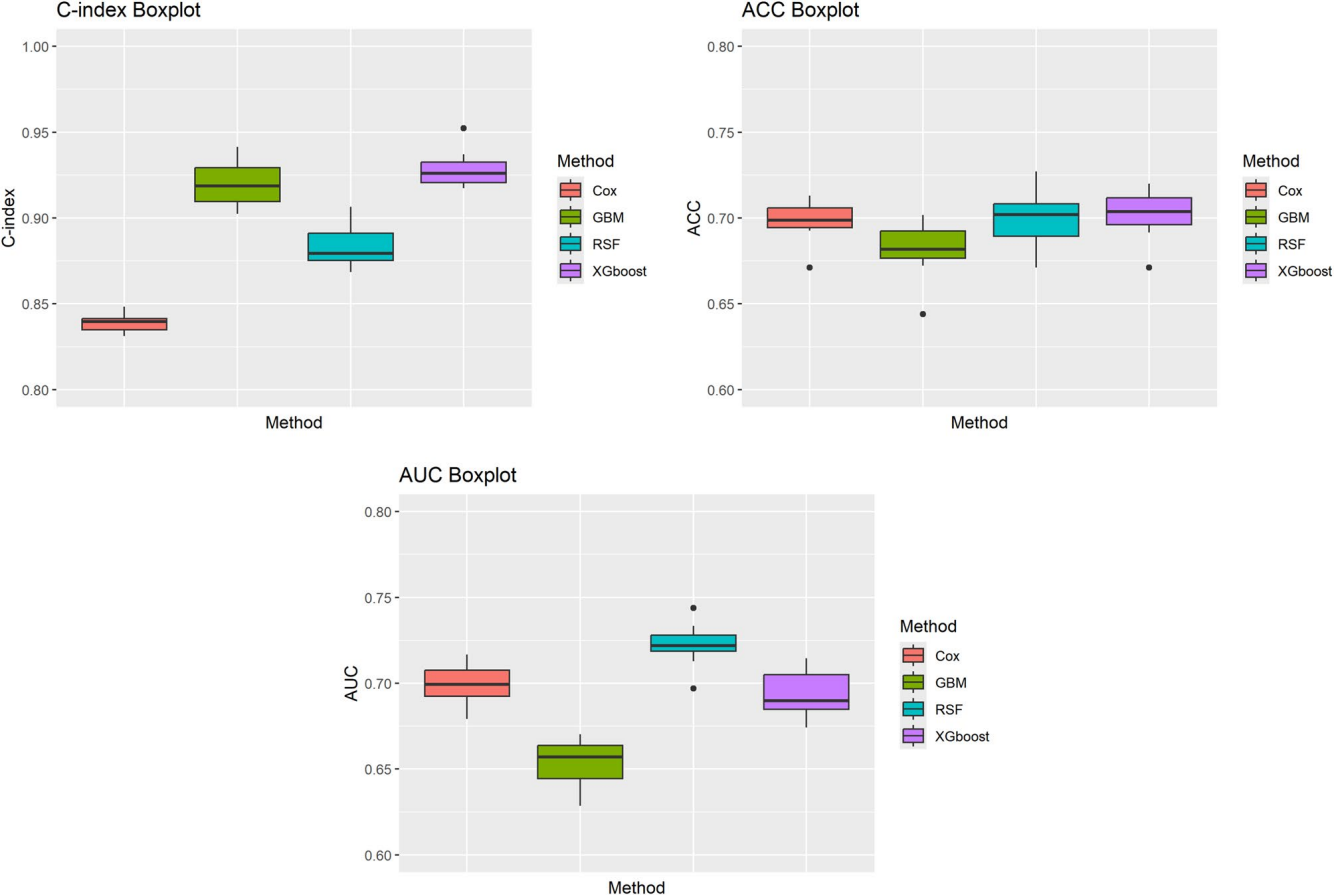
Boxplots of the C-index, ACC, and AUC values for the Cox model and machine learning methods

Figure 8 visually compares the performance of the models, with boxplots illustrating the variability and consistency of the results across different data splits. The boxplots for the C-index show that both XGBoost and RSF have narrower interquartile ranges, indicating that these models generalize well across the dataset. This is a crucial consideration in survival analysis, particularly in pharmaceutical applications where stability and consistency of model performance are necessary for clinical decision-making. Similar trends are observed for ACC and AUC, where XGBoost and RSF outperform the Cox model, while GBM exhibits greater variability in its performance, possibly due to its sensitivity to the data structure.

The superior performance of XGBoost can be attributed to its boosting mechanism, which refines predictions by iteratively improving on previous models. This allows XGBoost to capture complex relationships between features and outcomes, which is critical when predicting patient survival and response to pharmaceutical treatments. The consistent performance metrics, including the C-index and ACC, demonstrate XGBoost’s robustness and suitability for survival analysis, particularly in cases with complex interactions and non-linear relationships between features. Similarly, RSF shows remarkable stability across different data splits, highlighting its ability to handle censored survival data, which is often encountered in clinical research. RSF’s ensemble approach, which aggregates multiple decision trees, allows it to capture subtle patterns in time-to-event data without assuming proportional hazards, unlike the Cox model.

To assess whether these performance differences are statistically significant, we conduct non-parametric comparisons using the Friedman test followed by the Nemenyi post-hoc test. These tests are applied to the three evaluation metrics across all models. The results, as presented in Table 7, indicate that the performance differences, particularly in terms of the C-index and AUC, are statistically significant. The Friedman test reveals substantial overall differences among the models across all three metrics, with all p-values less than 0.05. Pairwise comparisons using the Nemenyi test show that XGBoost significantly outperforms the Cox model in terms of the C-index, with a p-value of $3.000 \times 10^{-6}$, and also demonstrates notable improvements over RSF and GBM in several metrics. For instance, the comparisons between Cox and GBM and between Cox and XGBoost yield significant differences in both C-index and AUC, supporting the superiority of machine learning methods. These statistical findings reinforce the observed ranking in performance metrics and confirm that the differences are not attributable to random variation.

**Table 7:** P-values from Friedman and Nemenyi tests across four models on three evaluation metrics.

Test Type	Model Comparison	C-index	ACC	AUC
Friedman Test (4 models)		$2.328 \times 10^{-6}$	0.021	$5.556 \times 10^{-6}$
Nemenyi Test	Cox vs. RSF	0.307	0.954	0.072
	Cox vs. GBM	0.002	0.160	0.029
	Cox vs. XGBoost	$3.000 \times 10^{-6}$	0.954	0.986
	RSF vs. GBM	0.226	0.046	$1.200 \times 10^{-6}$
	RSF vs. XGBoost	0.006	1.000	0.029
	GBM vs. XGBoost	0.508	0.046	0.072

In summary, machine learning methods like XGBoost and RSF outperform the Cox model in terms of prediction accuracy and generalizability, making them well-suited for complex datasets in pharmaceutical research. These methods provide a valuable tool for understanding patient survival and optimizing treatment regimens. As we move toward more personalized approaches in healthcare, the application of machine learning methods, particularly in survival analysis, offers a promising avenue for improving patient outcomes. This study demonstrates that machine learning methods not only complement traditional models like Cox but may eventually become the preferred approach in survival analysis, especially in the context of big data and personalized medicine. The results of the Friedman and Nemenyi tests further support these findings, confirming that the observed differences in predictive performance, particularly between XGBoost and Cox, are statistically significant across multiple evaluation metrics.

## Discussion

This study contributes a novel methodological integration that systematically compares penalized Cox regression models (SCAD, AEN, MCP) and ensemble-based machine learning algorithms (RSF, GBM, XGBoost) within a unified survival prediction framework tailored for sepsis. In addition to conventional penalization, we incorporate nonparametric information gainâ€“based feature selection and evaluate its impact across multiple modeling strategies. This hybrid approach bridges the gap between interpretability and predictive performance in sepsis prognosis and offers practical insights into individualized risk stratification. By employing advanced feature selection methods, we aimed to improve the predictive power and interpretability of these models. The focus on sepsis outcomes is particularly relevant to pharmaceutical research, where the goal is often to predict how patients will respond to treatments or interventions. Our analysis highlights the strengths and limitations of both traditional and modern predictive methods in handling high-dimensional clinical data. One key finding is that penalized feature selection methods like AEN and SCAD significantly enhance the Cox model’s performance by reducing overfitting and retaining clinically meaningful features. While AEN achieved a strong balance between complexity and predictive power, IG offered broader feature coverage, identifying additional relevant variables, though with slightly reduced accuracy.

Several of the top-ranking features identified by our models have strong clinical and biological relevance in the context of sepsis. For example, blood pressure variability reflects vascular tone and autonomic nervous system dysfunction, both of which are hallmarks of sepsis-related hemodynamic instability^41^. White blood cell count serves as a classic biomarker of infection and immune status^42^, while oxygen saturation is indicative of tissue hypoxia and has been correlated with mortality risk^43^. Low albumin levels are associated with impaired vascular integrity and fluid distribution^44^. Body mass index influences host metabolic and inflammatory responses^45^, and elevated BUN/creatinine ratios reflect renal involvement and can signal impending septic shock^46^. Incorporating such features into predictive models not only improves accuracy but also aligns with known pathophysiological mechanisms of sepsis.

In contrast to the Cox model, machine learning methods, particularly extreme gradient boosting (XGBoost), demonstrated consistently superior performance across all metrics, including C-index, accuracy, and area under the receiver operating characteristic curve (AUC). This superiority can largely be attributed to XGBoost’s ability to model complex, non-linear interactions between features, a capability that traditional regression models often lack. XGBoost’s performance highlights the importance of using methods capable of capturing intricate patterns in survival data, particularly when dealing with high-dimensional datasets as seen in sepsis studies. Similarly, random survival forests (RSF) and gradient boosting machine (GBM) also showed strong performance, especially in handling censored data and non-linearities, further emphasizing the advantages of ensemble methods in survival analysis. Their performance advantage is explained further below.

The superior performance of XGBoost and RSF over the Cox model is largely due to their non-parametric structure and ability to capture complex, nonlinear interactions between features. The Cox model relies on the proportional hazards assumption and linear log-risk functions, which may be violated in real-world sepsis data where time-to-event outcomes are influenced by intricate dependencies. XGBoost, with its iterative boosting framework and regularization techniques, can model these non-linearities more effectively. RSF, on the other hand, benefits from its ensemble of decision trees and use of log-rank splitting criteria, allowing it to accommodate right-censored data without making restrictive distributional assumptions. Furthermore, both models naturally handle missingness and interaction effects, which are common in high-dimensional clinical datasets. These algorithmic advantages make XGBoost and RSF more robust and better suited to the heterogeneity present in sepsis populations.

Our findings support the growing recognition that machine learning models, by leveraging advanced computational techniques, offer significant advantages in predictive modeling over traditional methods, especially when applied to complex clinical data. This is particularly relevant to the field of pharmaceutics, where precision in prediction is critical for optimizing drug therapies and improving patient outcomes. The results suggest that machine learning models, such as XGBoost and RSF, not only improve prediction accuracy but also offer greater flexibility in handling complex, multi-factorial clinical data. This positions these models as highly valuable tools in the development of predictive frameworks for personalized medicine. While ensemble-based models like XGBoost demonstrate strong predictive performance, one of their main drawbacks lies in limited interpretability compared to traditional approaches such as the Cox regression. This black-box characteristic can be a barrier in clinical applications, where transparency and trust are essential for adoption. To address this issue, model-agnostic tools, most notably SHapley Additive Explanations, have been introduced to help explain how specific features influence individual predictions^47^. Such techniques provide a useful compromise between predictive power and interpretability, which is especially important in high-stakes domains like personalized treatment and pharmacological decision-making.

While this study demonstrates the potential of machine learning models to enhance survival prediction in sepsis patients, several limitations should be acknowledged. First, one limitation of the current study is that the analysis was restricted to the MIMIC-III dataset. Future research will focus on validating the proposed framework using external datasets such as MIMIC-IV and eICU to evaluate the generalizability and robustness of the findings. Second, all models were developed and evaluated on the same dataset, raising concerns about external validity. Future work will focus on validating the proposed framework using independent, multi-center datasets such as MIMIC-IV and eICU. Third, the Cox model assumes proportional hazards, which may not hold across all covariates or patient subgroups. Although machine learning models like XGBoost and RSF address this issue by capturing nonlinear effects, their limited interpretability may hinder clinical adoption. Lastly, this study does not incorporate time-varying covariates or competing risks, which are relevant for modeling the clinical trajectory of sepsis. Future extensions will explore dynamic modeling strategies, such as multi-state models or cause-specific hazards frameworks, to improve clinical relevance.

## Conclusions

This study highlights the practical utility of modern machine learning (ML) methods in improving survival prediction for sepsis patients. Compared to the Cox proportional hazards model, ensemble-based models such as XGBoost, RSF, and GBM offer improved predictive performance and greater flexibility in capturing complex, non-linear relationships within complex clinical data. These advantages have direct implications for critical care: earlier identification of high-risk patients can enable timely interventions, such as prompt antibiotic administration or escalated monitoring, potentially improving survival outcomes.

Beyond predictive performance, the integration of feature selection techniques enhances interpretability by identifying clinically relevant indicators most associated with mortality risk. By emphasizing features such as lab markers and vital sign trends, our models support more targeted, individualized treatment strategies. In practice, these insights allow care teams to tailor therapy based on a patient’s predicted risk profile, advancing the goal of personalized medicine in sepsis care. By embedding these ML-based tools into clinical workflows, healthcare providers can move toward more anticipatory, dynamic, and patient-centered care, ultimately transforming therapeutic decision-making in the intensive care setting.

This study highlights the promise of machine learning in sepsis prognosis. Future research will explore hybrid modeling approaches that combine the interpretability of traditional models with the predictive power of machine learning techniques. In particular, blending machine learning algorithms with more traditional statistical methods could offer a way to capitalize on the strengths of both, providing robust, accurate predictions while maintaining the transparency needed for clinical decision-making. Additionally, unsupervised learning methods could be incorporated into future studies to identify latent patterns in sepsis progression and treatment outcomes, potentially uncovering new avenues for personalized treatment strategies. To further advance this line of research, future efforts could incorporate deep learningâ€“based survival models to evaluate potential performance gains and trade-offs in interpretability. Moreover, incorporating model interpretability tools such as SHAP could further support the clinical translation of complex models by clarifying the role of individual predictors.

The integration of feature selection methods such as AEN and SCAD into the Cox model substantially enhanced its predictive ability, underscoring the value of clinical feature prioritization. Nonetheless, our findings reaffirm that ensemble-based machine learning approaches, particularly XGBoost, are more capable of capturing complex interactions in sepsis data. In conclusion, while the Cox proportional hazards model remains a cornerstone of survival analysis, machine learning methods, especially XGBoost and RSF, offer a distinct advantage in terms of handling complex, high-dimensional clinical data. This study demonstrates that machine learning models not only complement traditional statistical approaches but may increasingly become the preferred tools for survival analysis in pharmaceutical research and personalized medicine. These methods provide healthcare professionals with more accurate and reliable tools for predicting patient outcomes, ultimately contributing to better patient care and more effective treatment strategies in critical care settings.

## Data Availability

The data presented in the study are available on PhysioNet at https://doi.org/10.13026/7vcr-e114.
